# Optimization of decompression angles in facial nerve decompression surgery: A decompression model

**DOI:** 10.1371/journal.pone.0340392

**Published:** 2026-01-30

**Authors:** Moeka Kanazawa, Fumihiro Mochizuki, Manabu Komori

**Affiliations:** 1 Department of Medicine, St. Marianna University School of Medicine, Kawasaki, Kanagawa, Japan; 2 Department of Otolaryngology, St. Marianna University School of Medicine, Kawasaki, Kanagawa, Japan; Hamad Medical Corporation, QATAR

## Abstract

Facial nerve decompression is a surgical procedure performed for severe facial nerve paralysis associated with conditions such as Bell’s palsy and Ramsay Hunt syndrome. Classical Western studies by Fisch first established the surgical principles of facial nerve decompression, providing the foundation for subsequent work on decompression extent and outcomes. However, the optimal extent of bony decompression of the facial nerve canal remains unclear, and a 180° removal of the surrounding bone has traditionally been performed based on empirical judgment. Nevertheless, more extensive bone removal may increase the risk of surgical complications. This study aimed to evaluate the relationship between the angle of bony decompression around the facial nerve canal and pressure reduction, in order to determine the optimal decompression angle. To achieve this, a simplified experimental model was employed to quantitatively evaluate the relationship between decompression angle and internal pressure reduction, providing mechanical insight into facial nerve decompression rather than clinical data. To evaluate this relationship, a decompression model was created, and pressure changes were measured at opening angles ranging from 30° to 180°. The results revealed that a 150° decompression provided a comparable reduction in pressure to that of a 180° decompression. These findings suggest that the extent of bone removal can be minimized while still achieving sufficient pressure reduction, potentially lowering the risk of nerve injury. We also observed significant pressure reduction at 30°, suggesting utility in regions where extensive bone removal is difficult. The finding that a 150° decompression produced an effect comparable to that of 180° is an important contribution toward improving surgical safety. Moving forward, we aim to refine the decompression model and conduct further investigations using more detailed angle settings, with the goal of establishing a practical surgical technique.

## Introduction

Facial nerve paralysis impairs voluntary facial movement—causing asymmetry, poor eye closure, and speech issues—and can markedly reduce quality of life [1–5].

Conservative treatment typically involves the administration of corticosteroids and antiviral agents. On the other hand, in cases where electroneurography (ENoG) conducted 10–14 days after onset shows less than 10% response, facial nerve decompression is indicated due to the poor prognosis [6]. This surgical procedure involves the removal of the bone surrounding the facial nerve to relieve nerve entrapment and improve blood flow, thereby promoting the recovery of nerve function. At our department, the procedure is often performed with preservation of the ossicular chain. When interference with the ossicles occurs, the incus is temporarily removed and repositioned after decompression.

Previous studies have suggested that early surgical decompression may improve the outcomes of severe facial nerve paralysis, particularly when performed within two weeks of onset [7,8]. However, there remains considerable debate regarding the optimal timing and extent of decompression. In contrast, no prospective studies have quantitatively evaluated the relationship between the degree of bony opening and decompression efficacy. Therefore, this study aimed to investigate the association between canal opening angles and internal pressure reduction using a physical model, providing a new perspective on optimizing the extent of decompression.

Traditionally, facial nerve decompression has involved opening 180° of the bony canal. However, extensive bone removal may increase the risk of complications, including nerve injury and hearing loss, due to the complexity of the procedure and the proximity to inner ear structures. Given the anatomically complex structure of the facial nerve and the limited extent of bone removal feasible at each segment, the development of surgical techniques that achieve maximal decompression with minimal bone removal is of great clinical value. Few studies quantify how opening angle relates to pressure reduction, underscoring the need for foundational work to optimize technique. This study aims to optimize the efficiency of decompression angles in facial nerve decompression surgery by measuring and evaluating the pressure reduction achieved at various opening angles, using a decompression model that simulates the facial nerve and its bony canal.

## Materials and methods

### Model construction

**Fig 1:**
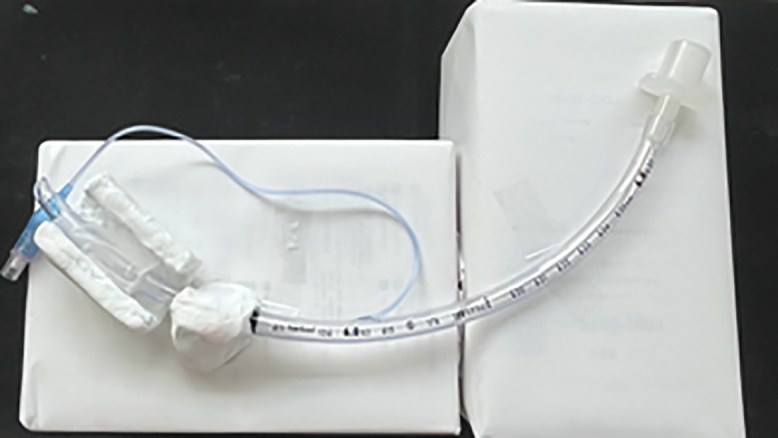
Top view of the decompression model. This model was created to evaluate the decompression effect based on the opening angle of the facial nerve canal in facial nerve decompression surgery. A tracheal tube with an inner diameter of 6.0 mm and an outer diameter of 8.2 mm (Shiley™, with TaperGuard™ cuff and stylet, Product No. 18760S; Medtronic Japan Co., Ltd.) was used to simulate the facial nerve. The canal model was positioned over the cuff portion of the tube. The photograph shows a top view of the entire model.

To evaluate the relationship between decompression angle and perineural pressure reduction in facial nerve decompression surgery, a decompression model was constructed. The decompression model is shown in Fig 1. As a facial nerve model, a tracheal tube with an inner diameter of 6.0 mm and an outer diameter of 8.2 mm (Shiley™, TaperGuard™ Cuffed, with stylet; Product No. 18760S; Medtronic Japan Co., Ltd.) was used. The facial nerve canal was simulated by covering a standard anesthesia breathing circuit (1.5 m, DAR Breathing System, Product No. 300P14347, Certification No. 220AABZX00156000) with a 7-mm-thick layer of clay. The clay-covered tube simulates the bony facial canal, while the tracheal tube represents the facial nerve.

**Fig 2:**
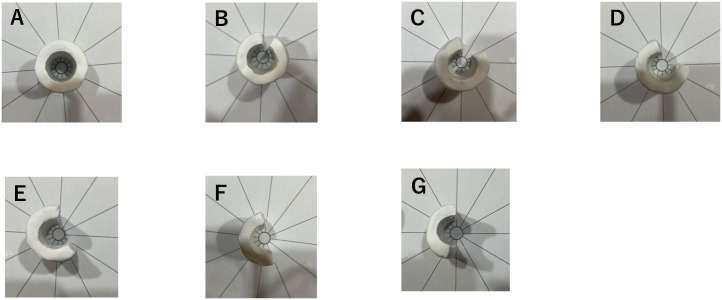
Facial nerve canal models created for each opening angle. **A Opening angle: 0°. B Opening angle: 30°. C Opening angle: 60°. D Opening angle: 90°. E Opening angle: 120°. F Opening angle: 150°. G Opening angle: 180**°. The facial nerve canal was simulated by covering a standard anesthesia circuit (1.5 m, DAR Breathing System, Product No. 300P14347, Certification No. 220AABZX00156000) with a 7-mm-thick layer of clay. The clay-covered section was assumed to represent the nerve canal, and external cuts were made at predetermined angles to create seven models with opening angles ranging from 0° (A) to 180° **(G)**. Each photograph shows a top view of the corresponding model.

The decompression model was configured with seven opening angles: 0°, 30°, 60°, 90°, 120°, 150°, and 180°. The clay-based nerve canal was incised at each specified angle using a ruler and protractor to create the decompression model. Fig 2 shows images of the nerve canal models at each decompression angle. A model in which the tracheal tube was not covered with clay and thus subjected to no external pressure was designated as the no-compression group.

### Colored water preparation and injection

**Fig 3:**
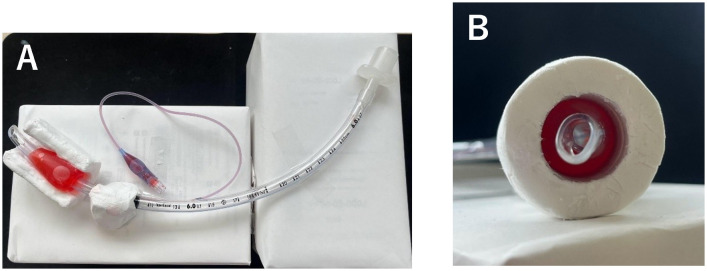
A. Top view of the decompression model with injected colored solution. The pressure inside the facial nerve canal was measured using a colored solution prepared by dissolving 0.1 g of red food coloring in 100 mL of normal saline. A volume of 6.6 mL of this solution was injected into the tracheal tube (simulating the nerve) to replicate internal pressure. In this model, the injection volume into the cuff was standardized at 6.6 mL. **B.** Cross-sectional view showing compression by the nerve canal model at an opening angle of 0°. Colored solution was injected into the tracheal tube simulating the nerve, and the entire outer surface was covered with the canal model to reproduce a constricted state at an opening angle of 0°. The image shows a lateral cross-sectional view of the model, in which the tracheal tube (representing the nerve) is seen to be circumferentially compressed by the canal. The red area represents the colored solution injected to simulate internal pressure.

To measure the pressure inside the nerve canal, colored water was used by dissolving 0.1 g of red food coloring in 100 mL of normal saline. A volume of 6.6 mL of colored water was injected into the tracheal tube to simulate internal pressure. In this model, the injection volume into the cuff was fixed at 6.6 mL. The injection volume of 6.6 mL was selected to achieve a mean internal pressure of approximately 28.9 cm H₂O under the no-compression condition, which falls within the physiologic range considered safe for tracheal cuff pressures that do not impair mucosal perfusion [9]. Although the present model simulates perineural rather than tracheal compression, this threshold was used as a practical reference to ensure that the applied pressure remained within a physiologically reasonable range. Volumes below 6 mL did not produce measurable compression within the canal model, whereas volumes above 8 mL caused unstable pressure fluctuations. Therefore, 6.6 mL was adopted as the optimal volume providing stable, reproducible, and physiologically relevant pressure conditions. Fig 3 shows the decompression model with colored water injected, while Fig 3B illustrates a cross-sectional view of the tracheal tube (simulating the nerve) being compressed by the surrounding nerve canal model. Only the cuff was surrounded by clay to simulate external compression. The cuff expands due to the hydrostatic pressure of the injected colored water, and the internal pressure is measured while its expansion is constrained by the clay-covered nerve canal model [10].

### Pressure measurement

**Fig 4:**
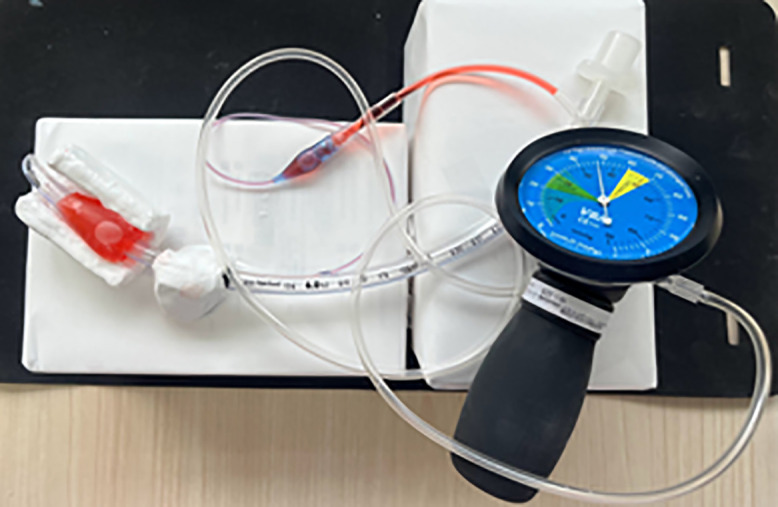
Decompression model during internal pressure measurement using the VBM Cuff Control Inflator. For each model with a different opening angle, internal pressure within the tracheal tube was measured using a VBM Cuff Control Inflator (VBM Medizintechnik GmbH, Germany; measurement range: 0–120 cm H₂O). The photograph shows a top-down view of the model with the colored solution injected, while the pressure gauge is connected to measure internal pressure.

For each decompression angle model, internal pressure was measured using a VBM Cuff Control Inflator (VBM Medizintechnik GmbH, Germany; measurement range: 0–120 cm H₂O). Measurements were repeated 21 times for each decompression angle, and the values from each trial were recorded. To minimize variation due to atmospheric pressure or operator-related factors, all measurements were performed under identical environmental conditions by the same operator. Fig 4 shows the decompression model during measurement using the VBM Cuff Control Inflator. The cuff is made of flexible PVC material and is designed to expand upon air injection. The internal pressure of the cuff when expanded within the clay-constrained environment indirectly reflects the magnitude of external compressive force. In this study, the internal cuff pressure was evaluated as a relative indicator of the decompression effect [10].

### Statistical analysis

Because some angle-wise data were non-normal by the Shapiro–Wilk test, we used nonparametric methods (see Supporting Information S1 Table for details). The Bonferroni correction was applied for multiple comparisons. We used software SPSS 28.0 (IBM Corp. Armonk, NY, USA) for the statistical analysis of this study.

### Ethics statement

This study did not involve human participants or animals. Therefore, ethical approval and informed consent were not required.

## Results

### Pressure measurement results

**Fig 5:**
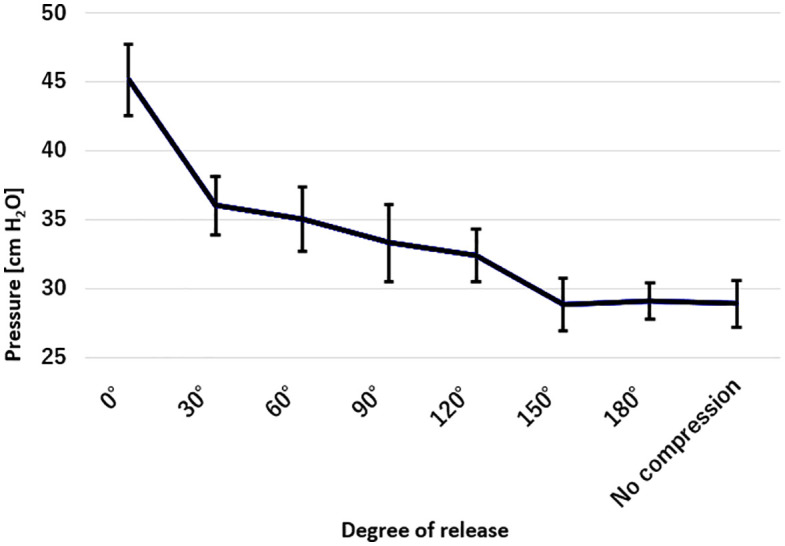
Relationship between the degree of canal opening and internal pressure. The vertical axis represents intraneural pressure (cm H₂O), and the horizontal axis indicates the degree of canal opening (0° to 180°), including the no-compression condition. A decreasing trend in intraneural pressure was observed with increasing degrees of canal opening. Notably, a marked reduction in pressure was seen between 0° and 30°, and pressures at 150° and beyond were comparable to those of the no-compression condition. Error bars indicate standard deviations (SD). The mean ± SD values were as follows: 0°, 45.1 ± 2.6; 30°, 36.0 ± 2.1; 60°, 35.0 ± 2.3; 90°, 33.3 ± 2.8; 120°, 32.4 ± 1.9; 150°, 28.8 ± 1.9; 180°, 29.1 ± 1.3; and no compression, 28.9 ± 1.7 cm H₂O.

Measurement of internal pressure at each decompression angle revealed a tendency for pressure to decrease as the angle increased. In particular, a marked decrease in pressure was observed between the 0° and 30° decompression angles. Table 1 presents the mean internal pressure ± standard deviation (cm H₂O) for each release angle, and Fig 5 shows the relationship between the degree of canal opening and internal pressure as a line graph. As shown in Fig 5, internal pressure significantly decreased with increasing canal opening angles. The largest drop was from 0° to 30°. At 150° and 180°, pressures matched the no-compression group (see Supporting Information S1 Fig A and B for details).

**Table 1:** Mean internal pressure ± standard deviation (cm H₂O) for each release angle.

	0°	30°	60°	90°	120°	150°	180°	No compression
**Average** **±SD[cmH**_**2**_ **O]**	45.1 ± 2.6	36 ± 2.1	35 ± 2.3	33.3 ± 2.8	32.4 ± 1.9	28.8 ± 1.9	29.1 ± 1.3	28.9 ± 1.7

The table presents the mean and standard deviation (SD) of internal pressure at each degree of canal opening and the no-compression condition. A decreasing trend in pressure was observed with increasing release angle, with a marked drop noted between 0° and 30°. Pressures at 150° and beyond were comparable to that of the no-compression group.

### Statistical analysis results

#### Test of normality.

The results of the Shapiro–Wilk test used to assess the normality of pressure values at each decompression angle are presented in S1 Table. Values were rounded to the fourth decimal place. In this analysis, a p-value > 0.05 was interpreted as indicating a normal distribution, whereas a p-value ≤ 0.05 was considered to indicate a deviation from normality. For five of the decompression angles, the data were assumed to follow a normal distribution (p > 0.05). However, at the 90° angle, the p-value was 0.008 (p < 0.05), indicating a deviation from normality. Therefore, non-parametric methods were used to compare the groups.

#### Multiple group comparisons.

The Friedman test was used to evaluate differences in pressure across decompression angles, yielding a statistically significant result (p = 6.86 × 10 ⁻ ^24^; test statistic = 125.03).

#### Pairwise comparisons.

Since the Friedman test revealed significant differences among groups, pairwise comparisons between each decompression angle and the no-compression group were performed using the Wilcoxon signed-rank test. The Bonferroni correction was applied, and the significance level was set at a Bonferroni-adjusted p-value < 0.007. The results are shown in S1 Fig A. A significant difference was found between the 0° decompression angle (45.1 ± 2.6 cm H₂O) and the no-compression group (28.9 ± 1.7 cm H₂O) (Bonferroni-adjusted p-value < 0.007). A significant difference was found between the 30° decompression angle (36.0 ± 2.1 cm H₂O) and the no-compression group (Bonferroni-adjusted p-value < 0.007). A significant difference was found between the 60° decompression angle (35.0 ± 2.3 cm H₂O) and the no-compression group (Bonferroni-adjusted p-value < 0.007). A significant difference was found between the 90° decompression angle (33.3 ± 2.8 cm H₂O) and the no-compression group (Bonferroni-adjusted p-value < 0.007). A significant difference was found between the 120° decompression angle (32.4 ± 1.9 cm H₂O) and the no-compression group (Bonferroni-adjusted p-value < 0.007). No significant difference was found between the 150° decompression angle (28.8 ± 1.9 cm H₂O) and the no-compression group (Bonferroni-adjusted p-value = 0.931). No significant difference was found between the 180° decompression angle (29.1 ± 1.3 cm H₂O) and the no-compression group (Bonferroni-adjusted p-value = 0.94). As described above, significant differences from the no-compression group were observed for decompression angles from 0° to 120°, whereas no significant differences were found at 150° and 180°.

Next, pairwise comparisons between each decompression angle and the 0° group were performed using the Wilcoxon signed-rank test. The Bonferroni correction was applied, and the significance level was set at a Bonferroni-adjusted p-value < 0.007. The results are shown in S1 Fig B significant difference was found between the 30° decompression angle (36.0 ± 2.1 cm H₂O) and the 0° group (45.1 ± 2.6 cm H₂O) (Bonferroni-adjusted p-value < 0.007). A significant difference was found between the 60° decompression angle (35.0 ± 2.3 cm H₂O) and the 0° group (Bonferroni-adjusted p-value < 0.007). A significant difference was found between the 90° decompression angle (33.3 ± 2.8 cm H₂O) and the 0° group (Bonferroni-adjusted p-value < 0.007). A significant difference was found between the 120° decompression angle (32.4 ± 1.9 cm H₂O) and the 0° group (Bonferroni-adjusted p-value < 0.007). A significant difference was found between the 150° decompression angle (28.8 ± 1.9 cm H₂O) and the 0° group (Bonferroni-adjusted p-value < 0.007). A significant difference was found between the 180° decompression angle (29.1 ± 1.3 cm H₂O) and the 0° group (Bonferroni-adjusted p-value < 0.007). A significant difference was found between the no-compression group (28.9 ± 1.7 cm H₂O) and the 0° group (Bonferroni-adjusted p-value < 0.007). As described above, all decompression angle groups showed Bonferroni-adjusted p-values < 0.007 when compared with the 0° group, indicating statistically significant differences in all cases.

## Discussion

This study quantitatively evaluated the relationship between the degree of canal opening and the reduction of nerve compression in facial nerve decompression surgery using a decompression model. The experimental results using the decompression model revealed that increasing the canal opening angle from 0° to 180° did not lead to a linear reduction in internal pressure. Instead, a marked pressure drop was observed at 30°, followed by a more gradual decrease at higher angles.

Notably, at a decompression angle of 150°, a pressure reduction comparable to that at 180° was observed. No statistically significant difference was found between the 150° and 180° decompression angles (p ≥ 0.007) These results suggest that in certain clinical scenarios—such as in patients with narrow anatomical spaces or elevated surgical risks—the conventional 180° bone removal may not be strictly necessary. Instead, a more conservative decompression angle may offer a safer yet effective alternative.

Previous reports have emphasized that extensive removal of the bone surrounding the facial nerve canal can reduce neural edema and promote early functional recovery [11]. In addition, animal studies using rat models have reported functional and histological changes corresponding to the severity of nerve compression injury. Specifically, no functional impairment or tissue damage was observed under low-pressure conditions (<100 mmHg), whereas moderate pressure (200–300 mmHg) resulted in reversible functional impairment and grade III axonal injury according to the Sunderland classification. Under high-pressure conditions (≥300 mmHg), irreversible functional impairment and severe grade V injury were observed [12]. However, investigations examining the effect of canal opening angle in facial nerve decompression surgery, as conducted in the present study, have not been previously reported. To date, no clinical studies have directly compared facial-nerve outcomes according to the degree or angular extent of decompression. Existing investigations have mainly focused on timing or surgical approach. For example, early transmastoid decompression with ossicular-chain preservation improved recovery in severe Bell’s palsy [13], whereas a comparative study found no additional benefit of surgical decompression over intensive medical therapy [14]. These findings suggest that clinical outcomes may depend more on surgical timing and indication than on the quantitative extent of decompression. The present experimental model was developed to explore this relationship under standardized conditions and to provide a foundation for future translational validation. Notably, even at the animal-model level, previous studies have focused mainly on the timing of decompression rather than on the quantitative extent or angle of bony opening. Therefore, the present study represents the first step toward quantitatively linking decompression angle to mechanical pressure reduction, providing a framework that future animal models may biologically validate. As discussed earlier, previous clinical studies, including those by Gantz BJ et al. and subsequent systematic reviews have suggested that early facial nerve decompression may improve recovery in severe Bell’s palsy [7,8]. However, no randomized controlled trials or large prospective cohort studies have directly compared different decompression extents or opening angles. Therefore, the evidence supporting the optimal degree of bony decompression remains limited, and the relationship between decompression angle and clinical outcome has not been prospectively established. The present model study was designed to address this specific gap by quantitatively evaluating the mechanical pressure–angle relationship under standardized conditions.

The results of this study also demonstrated that even a small canal opening of 30° led to a marked reduction in internal pressure. This suggests that even minimal bone removal can produce a clinically meaningful decompression effect. In the region from the first knee to the horizontal section, the extent of decompression is often anatomically limited, making sufficient opening technically challenging in some cases. In such anatomically constrained regions, the findings of this study suggest that even minimal decompression may achieve sufficient pressure reduction, providing useful insights for reconsidering surgical strategies [15]. In this region, the stapes and semicircular canals are in close proximity, and conventional 180° decompression may be difficult to perform or associated with a higher risk. In such circumstances, the finding that effective decompression can be achieved with a 150° opening—or even within the 30° to 120° range—is extremely valuable for enhancing flexibility in surgical strategies.

Recent literature has also emphasized that the extent of bony removal is a critical determinant of surgical safety. Limited extended epitympanotomy achieved adequate exposure without complications [16]. Conversely, extensive decompression via the middle fossa approach has been associated with a higher risk of complications such as hearing loss, cerebrospinal fluid leakage, and temporal lobe injury [8]. These findings suggest that limiting the extent of bone removal can maintain surgical safety while achieving adequate decompression. Based on these findings, the present model experiment demonstrated that limiting the bony opening around the facial nerve to 150°, rather than the conventional 180°, produced a comparable decompression effect. Therefore, future studies should investigate whether restricting the canal opening to 150° and reducing the extent of bone removal could help decrease the risk of postoperative complications.

From an anatomical standpoint, a canal opening of approximately 150° is technically feasible and safe within the tympanic (horizontal) segment, especially between the first genu and the horizontal portion, where the working space is relatively wide and the risk to inner-ear structures is minimal. These findings support the clinical interpretation that 150° decompression provides sufficient pressure reduction while minimizing unnecessary bone removal. Moreover, the present results demonstrated that measurable pressure reduction was already achieved at a 30° canal opening. This observation suggests that even limited decompression can provide partial pressure relief and may help reduce the need for risky or extensive procedures in constrained areas. It also expands the range of intraoperative options according to individual anatomy and the surgeon’s proficiency. From the educational perspective, the present findings highlight the importance of learning to perform decompression within a safe and feasible range appropriate to one’s technical level, under the supervision of experienced surgeons, which may serve as a rational basis for progressive skill development and simulation training.

Although our physical model does not replicate the viscoelastic or vascular properties of living tissue, animal studies have demonstrated that mechanical decompression facilitates both functional and histological improvement of the facial nerve. In a rat model of complete facial paralysis, decompression performed one or three weeks after induced compression led to significantly better functional and electrophysiological outcomes than no decompression, with no significant difference between early and delayed decompression. Histological analysis showed reduced fibrosis and preservation of myelin structure in the decompressed group [17]. These findings suggest that the pressure reduction observed in this model may reflect fundamental physiological mechanisms involved in nerve recovery following decompression. Previous clinical studies have demonstrated that surgical decompression medial to the geniculate ganglion can significantly improve facial nerve recovery when performed within two weeks after onset [7]. This finding underscores the clinical relevance of timely and adequate decompression, aligning with the pressure–relief relationship observed in our model.

However, this study has several limitations. First, the endotracheal tube used to simulate the nerve differs from biological nerves in its physical properties, and therefore may not fully replicate clinical pressure dynamics. Additionally, while the decompression model used in this study was simplified to enhance reproducibility and generalizability, it does not fully reflect the anatomical complexity of the human skull. This simplified structure was intentionally adopted because the primary purpose of this study was to perform repeated and reproducible measurements under identical decompression angles and standardized conditions. Since the interface between the PVC cuff and the surrounding clay exhibits lower friction and higher elasticity than the actual bone–nerve interface in vivo, the degree of pressure reduction observed in this model may be slightly overestimated compared with real clinical decompression. Nevertheless, as all measurements were conducted under identical experimental conditions, we believe that the relative comparison among different opening angles remains reliable and meaningful.

In addition, the validity of using a simplified physical system to investigate nerve compression and decompression dynamics is supported by recent preclinical studies. For example, Daeschler et al. developed a sensor-based nerve compression model combining synthetic and ex vivo components to quantify mechanical pressure under controlled conditions [18]. Their findings demonstrated that such simplified, reproducible setups can meaningfully represent the biomechanical behavior of nerve compression, even though they do not replicate the full histological or vascular complexity of in vivo tissues. In the present study, we prioritized high reproducibility and statistical reliability by performing repeated measurements at 30° intervals under standardized conditions. A major advantage of this approach is that it allows multiple trials without the need for expensive biological materials or high-cost experimental facilities.

Based on the present dataset, future studies may expand this framework to include biologically integrated elements—such as cadaveric or ex vivo temporal bone models—to explore the detailed differences between 150° and 180° openings, or between 0° and 30°, under more physiological conditions. This conceptual consistency supports the use of our physical model as a foundational tool for understanding the pressure–angle relationship in facial nerve decompression. Furthermore, the model was designed to reproduce the overall trend of pressure changes associated with various canal opening angles, thereby providing a controlled and educational framework for understanding how decompression extent influences pressure relief, even though absolute pressure values may differ from in vivo conditions. In the future, studies incorporating materials that more closely mimic the physical properties of nerve tissue, as well as models with progressively refined anatomical structures, are warranted. Decompression was performed at 30° intervals to maintain a simple and reproducible setup. This design may limit the resolution of pressure measurements and overlook subtle transitions between effective and maximal decompression. Future studies assessing intermediate angles (e.g., 135°, 165°) may refine the quantitative understanding of decompression effects. The decompression model was also designed to resemble actual anatomical structures. However, a limitation of the model is that it does not replicate actual nerve edema or vascular impairment observed in vivo. Rather, the model was intentionally designed to simply reproduce the trend between canal opening angles and pressure changes, making it suitable for evaluating surgical techniques and for educational applications [10].

In addition, while the actual luminal diameter of the facial nerve varies depending on the anatomical location, it was kept constant in this model. For example, the labyrinthine segment has the narrowest luminal diameter and is the most prone to constriction [19]. On the other hand, accurately reproducing the spatial relationship between the nerve and the bony canal remains challenging in this model.

Furthermore, in this study, canal opening angles were set at 30° intervals to capture overall trends; therefore, subtle changes at intermediate angles such as 135° or 165° were not evaluated. In addition, the smallest canal opening angle examined in this study was 30°, at which a marked reduction in nerve compression was observed.

In summary, this study represents the first quantitative approach to optimizing the degree of canal opening in facial nerve decompression and provides a novel perspective for refining conventional surgical strategies.

## Conclusions

In this study, we quantitatively investigated the relationship between the degree of canal opening and the reduction of nerve compression in facial nerve decompression using a decompression model. As a result, it was found that a canal opening angle of 150° achieved a reduction in internal pressure comparable to that of the conventional standard of 180°. Furthermore, a notable reduction in internal pressure was observed even at the relatively narrow opening angle of 30°, suggesting that minimal bone removal may substantially alleviate nerve compression. These findings suggest that rather than blindly adhering to the conventional standard of 180° decompression, it is important to consider individual anatomical conditions and surgical risks in order to select a more efficient and safer decompression strategy.

These findings provide preliminary mechanical insight into the relationship between decompression extent and pressure reduction based on an experimental model. Further validation through cadaveric and clinical studies will be necessary to confirm the clinical applicability of these results. An opening angle of 150°, which provides a comparable reduction in internal pressure to 180° while minimizing the extent of bony removal, may represent a useful reference for refining decompression techniques and may contribute to the future standardization of surgical techniques. Further research is warranted to establish more clinically applicable guidelines.

## Supporting information

S1 Fig Statistical analysis results. The Friedman test was used to evaluate differences in pressure across decompression angles, yielding a statistically significant result (p = 6.86 × 10 ⁻ ²⁴; test statistic = 125.03). **A Comparison of internal pressure between the no-compression group and each degree of canal opening (Wilcoxon signed-rank test with Bonferroni correction).** Since the Friedman test revealed significant differences among groups, pairwise comparisons between each decompression angle and the no-compression group were performed using the Wilcoxon signed-rank test. The Bonferroni correction was applied, and the significance level was set at a Bonferroni-adjusted p-value < 0.007. The results are shown in this Fig. A significant difference was found between the 0° decompression angle (45.1 ± 2.6 cm H₂O) and the no-compression group (28.9 ± 1.7 cm H₂O) (Bonferroni-adjusted p-value < 0.007). A significant difference was found between the 30° decompression angle (36.0 ± 2.1 cm H₂O) and the no-compression group (Bonferroni-adjusted p-value < 0.007). A significant difference was found between the 60° decompression angle (35.0 ± 2.3 cm H₂O) and the no-compression group (Bonferroni-adjusted p-value < 0.007). A significant difference was found between the 90° decompression angle (33.3 ± 2.8 cm H₂O) and the no-compression group (Bonferroni-adjusted p-value < 0.007). A significant difference was found between the 120° decompression angle (32.4 ± 1.9 cm H₂O) and the no-compression group (Bonferroni-adjusted p-value < 0.007). No significant difference was found between the 150° decompression angle (28.8 ± 1.9 cm H₂O) and the no-compression group (Bonferroni-adjusted p-value = 0.931). No significant difference was found between the 180° decompression angle (29.1 ± 1.3 cm H₂O) and the no-compression group (Bonferroni-adjusted p-value = 0.94). As described above, significant differences from the no-compression group were observed for decompression angles from 0° to 120°, whereas no significant differences were found at 150° and 180°. A Friedman test revealed significant differences among groups, prompting pairwise comparisons between the no-compression condition and each release angle using the Wilcoxon signed-rank test. Bonferroni correction was applied, with the adjusted significance level set at p < 0.007. Significant differences were observed between the no-compression group (28.9 ± 1.7 cm H₂O) and release angles from 0° (45.1 ± 2.6 cm H₂O) to 120° (32.4 ± 1.9 cm H₂O) (p < 0.007). However, no significant differences were found for 150° (28.8 ± 1.9 cm H₂O) and 180° (29.1 ± 1.3 cm H₂O) (p > 0.007). The vertical axis indicates intraneural pressure (cm H₂O), and the horizontal axis represents the degree of canal opening. **B Comparison of internal pressure between 0° and all other release angles (Wilcoxon signed-rank test with Bonferroni correction).** Pairwise comparisons between each decompression angle and the 0° group were performed using the Wilcoxon signed-rank test. The Bonferroni correction was applied, and the significance level was set at a Bonferroni-adjusted p-value < 0.007. The results are shown in this Fig significant difference was found between the 30° decompression angle (36.0 ± 2.1 cm H₂O) and the 0° group (45.1 ± 2.6 cm H₂O) (Bonferroni-adjusted p-value < 0.007). A significant difference was found between the 60° decompression angle (35.0 ± 2.3 cm H₂O) and the 0° group (Bonferroni-adjusted p-value < 0.007). A significant difference was found between the 90° decompression angle (33.3 ± 2.8 cm H₂O) and the 0° group (Bonferroni-adjusted p-value < 0.007). A significant difference was found between the 120° decompression angle (32.4 ± 1.9 cm H₂O) and the 0° group (Bonferroni-adjusted p-value < 0.007). A significant difference was found between the 150° decompression angle (28.8 ± 1.9 cm H₂O) and the 0° group (Bonferroni-adjusted p-value < 0.007). A significant difference was found between the 180° decompression angle (29.1 ± 1.3 cm H₂O) and the 0° group (Bonferroni-adjusted p-value < 0.007). A significant difference was found between the no-compression group (28.9 ± 1.7 cm H₂O) and the 0° group (Bonferroni-adjusted p-value < 0.007). As described above, all decompression angle groups showed Bonferroni-adjusted p-values < 0.007 when compared with the 0° group, indicating statistically significant differences in all cases. Pairwise comparisons between the 0° group and each release angle group were performed using the Wilcoxon signed-rank test. Bonferroni correction was applied, and the significance level was set at p < 0.007. Significant differences (p < 0.007) were observed between the 0° group (45.1 ± 2.6 cm H₂O) and all other groups, ranging from 30° (36.0 ± 2.1 cm H₂O) to 180° (29.1 ± 1.3 cm H₂O), as well as the no-compression group (28.9 ± 1.7 cm H₂O). The vertical axis represents intraneural pressure (cm H₂O), and the horizontal axis represents the degree of canal opening.
(ZIP)

S1 Table Shapiro-Wilk test p-values for normality of internal pressure at each release angle. This table presents the p-values from Shapiro-Wilk tests evaluating the normality of intraneural pressure distributions at each release angle. Values were rounded to the fourth decimal place. A p-value > 0.05 was considered consistent with a normal distribution, whereas p ≤ 0.05 indicated deviation from normality. Normality was assumed for all angles except for 90°, where p = 0.008 suggested a non-normal distribution.
(XLSX)
